# Associations between systemic inflammation indicators and nonalcoholic fatty liver disease: evidence from a prospective study

**DOI:** 10.3389/fimmu.2024.1389967

**Published:** 2024-06-24

**Authors:** Hao Gong, Qida He, Lili Zhu, Zhaolong Feng, Mengtong Sun, Jingting Jiang, Xiaofeng Yuan, Yueping Shen, Jia Di

**Affiliations:** ^1^ Infection Management Department, The First People’s Hospital of Changzhou, Changzhou, China; ^2^ Department of Epidemiology and Biostatistics, School of Public Health, Medical College of Soochow University, Suzhou, China; ^3^ Tumor Biological Diagnosis and Treatment Center, The First People’s Hospital of Changzhou, Changzhou, China; ^4^ Department of Spine Surgery, The First People’s Hospital of Changzhou, Changzhou, China

**Keywords:** nonalcoholic fatty liver disease, hepatic steatosis, systemic inflammation, UK Biobank, prospective studies

## Abstract

**Background:**

Although inflammation has been linked to nonalcoholic fatty liver disease (NAFLD), most studies have focused only on a single indicator, leading to inconsistent results. Therefore, a large prospective study that includes a variety of well-documented single and composite indicators of inflammation is needed. This study aimed to thoroughly investigate the potential associations between different systemic inflammatory indicators and NAFLD in the UK Biobank cohort.

**Methods:**

After excluding ineligible participants, 378,139 individuals were included in the study. Associations between systemic inflammatory indicators and hepatic steatosis were assessed using multivariate logistic regression. The relationships between systemic inflammatory indicators and nonalcoholic fatty liver disease were analysed using Cox proportional hazards models, and nonlinear associations were investigated using restricted cubic splines.

**Results:**

According to the cross-sectional analysis, systemic inflammatory indicators significantly correlated with hepatic steatosis. Over a median follow-up of 13.9 years, 4,145 individuals developed NAFLD. After sufficient adjustment for confounding factors, CRP levels were found to be nonlinearly positively associated with NAFLD risk (*P*<0.001), representing the strongest correlation among the tested relationships; lymphocyte count and the LMR showed an L-shaped correlation; monocyte count and neutrophil count showed a linear positive correlation (all *P*< 0.001); and the NLR, PLR, and SII showed a U-shaped correlation (all *P*<0.001).

**Conclusions:**

Multiple systemic inflammatory indicators are strongly associated with the development of NAFLD, and aggressive systemic inflammation management may have a favourable impact on reducing the burden of NAFLD; further randomized controlled studies are needed.

## 1 Introduction

Nonalcoholic fatty liver disease (NAFLD), a prevalent chronic liver disease characterized by the excessive accumulation of lipids in the liver, is a major cause of end-stage liver disease, affecting approximately 30% of the global population (1). It ranges from benign steatosis to inflammatory nonalcoholic steatohepatitis (NASH) (2), which can lead to cirrhosis and hepatocellular carcinoma, thereby significantly increasing morbidity and mortality (3, 4). Because of the increasing incidence of obesity and metabolic syndrome, the prevalence of NAFLD worldwide is considerably greater than previously estimated and is continuing to increase at an alarming rate (5, 6), resulting in an increasing social and financial burden (7). Prevention of NAFLD has become an important public health challenge globally.

Several chronic diseases are influenced by systemic inflammation, which is caused by the release of proinflammatory cytokines and persistent innate immune system activation (8–12). The development of NAFLD is characterized by inflammation, and hepatic steatosis and fibrosis are significantly influenced by the migration of circulating inflammatory cells and the overexpression of inflammatory mediators (13). Therefore, clinical parameters of plasma cytokines and inflammation can be used as a new strategy to monitor the progression of NAFLD (14). The systemic immune-inflammation index (SII) is an integrated and novel inflammatory biomarker that can reflect the local immune response and systemic inflammation in the whole human body (15–17). A recent study confirmed that the SII is positively associated with increased hepatic steatosis in US adults (18). Although there is growing evidence that inflammation dysregulation plays a part in NAFLD, most related studies have focused only on a single biomarker and have drawn controversial conclusions (19–22). Therefore, a thorough investigation into the relationship between systemic inflammation and NAFLD is needed.

To address these limitations, this study aimed to explore the linear and nonlinear relationships between eight systemic inflammatory indicators and hepatic steatosis and NAFLD fetching the UK Biobank (UKB), which is a large-scale population-based prospective cohort study.

## 2 Methods

### 2.1 Study population

The UKB recruited over 500,000 participants aged 37–73 years from the general UK population between 2006 and 2010 (23). Through questionnaires and physical measurements, all participants provided clinical data, physical measurements, baseline blood samples, and relevant electronic health information. Links to national health-related databases allowed for the tracking and follow-up of each participant’s health results. Participants provided written, informed consent at baseline. Two separate substudies were constructed: a cross-sectional study of patients with hepatic steatosis at baseline [judged based on the hepatic steatosis index (HSI) progression] and a prospective cohort study in which participants with hepatic steatosis were excluded at baseline to investigate the prevalence of NAFLD. This study used the UKB resource with application number 68136 and complied with the Strengthening Reporting of Observational Studies in Epidemiology (STROBE) reporting standards.

**Figure 1:**
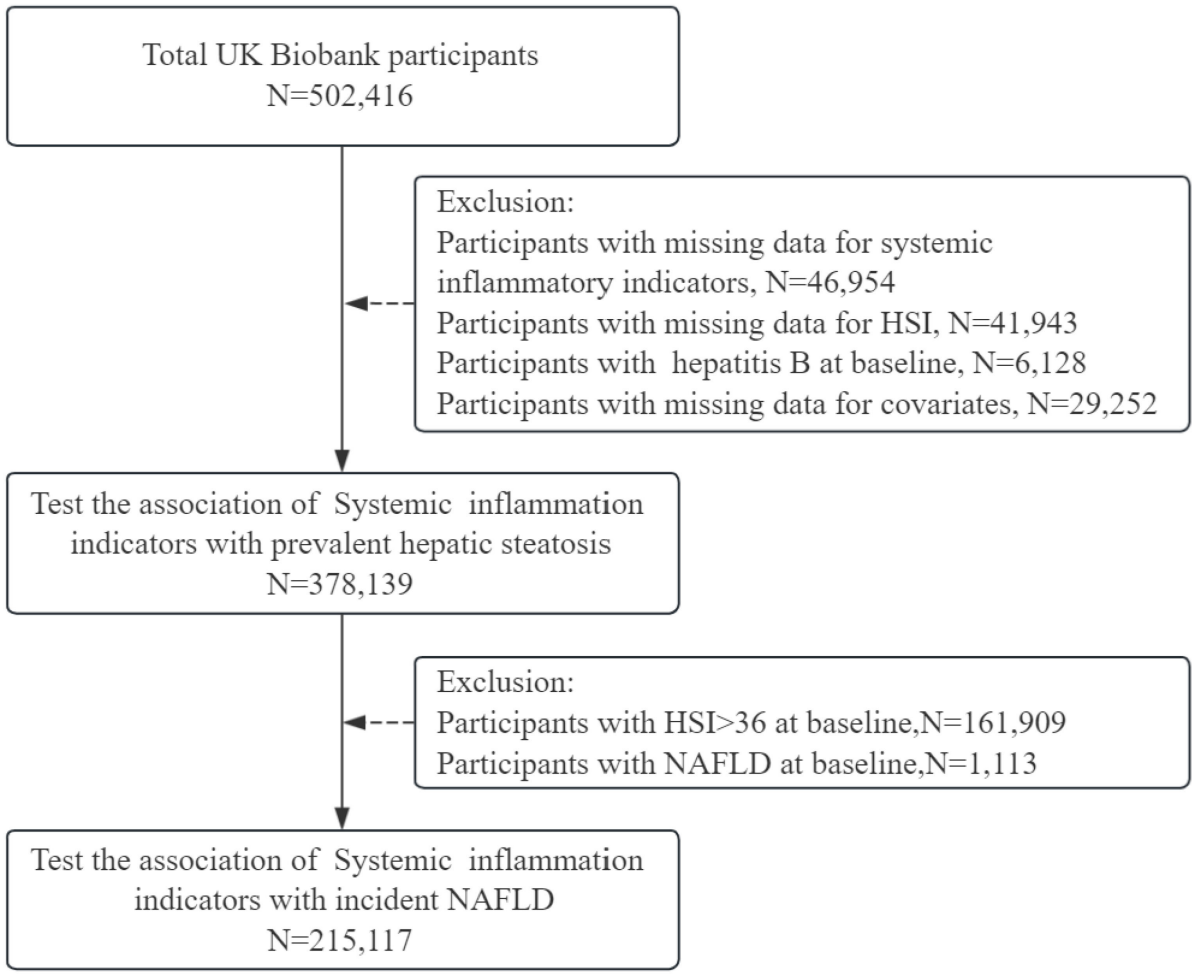
Flowchart of participants included in the analyses.

Participants were excluded if they had missing data on systemic inflammatory indicators (N=46,954), HSI (N=41,943), or covariates (N = 29,252). Participants with a history of hepatitis B (judged by the presence of HBC antigen) at baseline were also excluded (N = 6,128). Consequently, this cross-sectional study ultimately included 378,139 participants. In the prospective cohort study, participants with a baseline HSI > 36 (N =161,909) and those with NAFLD (N =1,113) were excluded, and a total of 215,117 participants were enrolled (**Figure 1**).

### 2.2 Systemic inflammation indicators

Peripheral blood samples from UKB members were examined using the Beckman Coulter LH 750 haematology analyser in the UKB laboratory within 24 hours of collection. Thirty-one parameters were recorded by the gadget, and it was possible to extract specific blood cell populations from them. Baseline count data for neutrophils, monocytes, lymphocytes, and platelets (operating range 0.00–900.00 × 10^9 cells/L) were extracted, and values for four composite inflammatory indices were calculated: the LMR, NLR, PLR, and SII. The calculations were as follows: LMR= lymphocytes/monocytes, NLR= neutrophils/lymphocytes, PLR = platelets/lymphocytes, and SII = (neutrophils * platelets)/lymphocytes. Since other studies have shown that these four ratios can accurately predict inflammatory states under a variety of circumstances, we took them into account in our analysis (11, 18, 24–26). Serum C-reactive protein (CRP) was also measured in this investigation using the Beckman Coulter AU5800 high-sensitivity immunoturbidimetric method.

### 2.3 Outcome ascertainment

In the cross-sectional analysis,we attempted to explore the relationship between systemic inflammatory indicators and all stages of NAFLD (from hepatic steatosis to severe NAFLD). The HSI was computed using baseline data to ascertain whether hepatic steatosis was present at the time of the participant’s initial follow-up enrolment. The HSI was calculated using the following formula: HSI = 8 × (alanine aminotransferase (ALT)/aspartate aminotransferase (AST) ratio) + body mass index (BMI) + 2 (if the participant has diabetes) + 2 (if the participant is female) (27). A significant correlation between the degree of hepatic steatosis and the HSI was previously reported, and an HSI > 36 is typically regarded as indicative of NAFLD (28, 29). Consequently, the cut-off points in this study for determining the presence of hepatic steatosis was set at HSI = 36, and any subjects whose HSI was greater than 36 were not included in the survival analyses.

In the prospective study, the primary outcome was severe NAFLD. Severe NAFLD was defined as hospitalisation or death due to NAFLD or NASH (30–32), according to the International Classification of Diseases, 10th edition (ICD-10) and the latest expert group consensus statement (33), NAFLD is defined as ICD-10 K76.0 (fatty liver, not elsewhere classified) and K75.8 (NASH, other specified inflammatory liver diseases).

### 2.4 Covariates

By utilizing the participants’ date of birth and initial assessment, the mean baseline age of the study participants was established at the outset. Sex was self-reported by the participants during the baseline evaluation. The classification of educational attainment was as follows: those who completed college or university were classified as having a university degree; those who completed middle school or an O level/GCSE or equivalent were classified as having an O level/AS level or equivalent; and those who completed any other type of education were classified as not fitting into any of the aforementioned categories. BMI was computed using the standard formula, which divides weight (in kilograms) by height (in metres squared), which were measured during the initial assessment. The Townsend Deprivation Index was used to determine economic status; higher values indicated a higher level of poverty (34). Based on self-reports, race was categorized as white or other. Three categories were used to classify self-reported smoking status: ‘never smoked’, ‘former smoker’, and ‘current smoker’. Based on participants’ answers to a question regarding the number of days per week that they engaged in more than 10 minutes of moderate physical activity, physical activity levels were classified as light, moderate, or high. Additionally, baseline information was gathered on high triglycerides, low HDL, hypertension, and diabetes. A fasting blood glucose level of 5.6 mmol/l or a self-reported physician diagnosis of diabetes was used to define hyperglycaemia/diabetes. The criteria for hypertension/high blood pressure were self-reported physician-diagnosed hypertension or a systolic blood pressure of at least 130 mm Hg and/or a diastolic blood pressure of at least 85 mm Hg. Low HDL cholesterol was defined as < 1.0 mmol/l for men and < 1.3 mmol/l for women, while high triglycerides were defined as levels ≥ 1.7 mmol/l (35).

### 2.5 Statistical analyses

Categorical variables are expressed as proportions, and continuous variables are expressed as the mean ± standard deviation. Using multivariate logistic regression analysis, the odds ratio (OR) and 95% confidence interval (95% CI) were used to evaluate the correlation between the systemic inflammation indicators and hepatic steatosis in a cross-sectional study. Using Cox proportional hazards models, the hazard ratio (HR) and 95% CI were used to estimate the associations between systemic inflammatory indicators and NAFLD risk in this prospective study. The proportional hazard assumption was checked using Schoenfeld residuals.

Three models were constructed in this study: Model 1 included no adjustments for covariates; Model 2 included adjustments for sex, age, race, education, BMI, Townsend deprivation index, smoking status, and physical activity; and Model 3 included additional baseline-related diseases such as high triglycerides, low HDL, hypertension, and diabetes. The possible nonlinear effects of systemic inflammatory status on NAFLD were evaluated using a restricted cubic spline with four knots positioned at the 5th, 35th, 65th, and 95th percentiles following the removal of outliers outside of the 1^th^-99^th^ percentile (36). Systemic inflammation indicators were used as continuous exposure variables. To make it easier to compare the relationships between different systemic inflammation indicators and NAFLD under various models, the systemic inflammation indicators were further classified into five categories based on the references, accounting for the normal reference range, data distribution, and easy-to-understand numbers (12). Subgroup analyses were carried out to investigate the relationships between systemic inflammatory indicators and NAFLD in various subgroups. Age (<60/≥60 years), sex (male/female), and BMI (<25/≥25) were stratified. Sensitivity analyses were performed to mitigate any potential effects of reverse causality: individuals with events in the first 2 years or the first 5 years of follow-up were excluded, and frequency of alcohol consumption was additionally adjusted. SAS version 9.4 and R version 4.3.0 were utilized for the analysis. A two-tailed *P* value less than 0.05 was considered to indicate statistical significance.

## 3 Results

### 3.1 Baseline characteristics of participants

Based on the presence of hepatic steatosis, baseline traits and systemic inflammatory indicators were examined in 378,139 individuals (**Table 1**). At baseline, 161,909 of the total participants had hepatic steatosis. Hepatic steatosis patients were more likely to be older, male, less educated, engage in less daily exercise, have lower levels of deprivation, have a higher BMI, have a higher systolic blood pressure, have a greater prevalence of cigarette smoking, and have a higher prevalence of metabolic syndrome than the 216,230 participants in the control group.

**Table 1:** Baseline cohort characteristics.

Characteristic	No hepatic steatosis (n=216,230)	Hepatic steatosis (n=161,909)	*P* value
**Age (years)**	56.40 ± 8.21	56.77 ± 7.91	<0.0001
**Female, *n* (%)**	118,773 (54.93%)	84,276 (52.05%)	<0.0001
**White, race, *n* (%)**	204,858 (94.74)	151,814 (93.77%)	<0.0001
**Townsend deprivation index**	-1.51 ± 2.98	-1.10 ± 3.15	<0.0001
**Educational level, *n* (%)**			<0.0001
College degree	78,808 (36.45%)	43,298 (26.74%)	
Middle school graduate	70,337 (32.53%)	52,980 (32.72%)	
None of the above	67,085 (31.02%)	65,631 (40.54%)	
**Body mass index (kg/m²)**	24.48 ± 2.46	31.32 ± 4.23	<0.0001
**Smoking status, *n* (%)**			<0.0001
Never	122,333 (56.58%)	84,169 (51.99%)	
Previous	70,269 (32.50%)	61,673 (38.09%)	
Current	23,628 (10.93%)	16,067 (9.92%)	
**Physical activity, *n* (%)**			<0.0001
Light	75,572 (34.82%)	71,484 (43.93%)	
Moderate	53,360 (24.59%)	36,690 (22.55)	
High	88,110 (40.50%)	54,546 (33.52%)	
Disease history at baseline, *n* (%)			
Hypertension	79,027 (36.55%)	99,562 (61.49%)	<0.0001
Diabetes	19,712 (9.12%)	44,036 (27.20%)	<0.0001
High triglycerides	61,274 (28.34%)	90,245 (55.74%)	<0.0001
Low HDL	26,019 (12.03%)	49,557 (30.61%)	<0.0001
**Diastolic blood pressure**	80.14 ± 10.43	85.06 ± 10.35	<0.0001
**Systolic blood pressure**	137.17 ± 19.77	143.27 ± 18.89	<0.0001
Systemic inflammation indicators			
C-reactive protein (CRP, mg/L)	1.94 ± 3.90	3.46 ± 4.69	<0.0001
Platelet count (10^9 cells/L)	250.49 ± 58.89	255.29 ± 60.74	<0.0001
Lymphocyte count (10^9 cells/L)	1.89 ± 1.32	2.07 ± 0.85	<0.0001
Monocyte count (10^9 cells/L)	0.46 ± 0.32	0.50 ± 0.22	<0.0001
Neutrophil count (10^9 cells/L)	4.10 ± 1.40	4.41 ± 1.42	<0.0001
Systemic immune-inflammation (SII)	599.02 ± 359.59	596.32 ± 370.83	<0.0001
Neutrophil-to-lymphocyte ratio (NLR)	2.38 ± 1.21	2.33 ± 1.29	<0.0001
Platelet-to-lymphocyte ratio (PLR)	146.11 ± 60.28	135.29 ± 63.34	<0.0001
Lymphocyte-to-monocyte ratio (LMR)	4.60 ± 3.74	4.67 ± 4.96	<0.0001
Alanine aminotransferase (U/L)	18.93 ± 8.56	29.61 ± 17.41	<0.0001
Aspartate aminotransferase (U/L)	25.19 ± 9.28	27.56 ± 11.10	<0.0001
Triglycerides	1.48 ± 0.82	2.09 ± 1.13	<0.0001
Glucose	4.93 ± 0.82	5.39 ± 1.58	<0.0001
HDL cholesterol	1.55 ± 0.39	1.32 ± 0.33	<0.0001

### 3.2 Cross-sectional analysis

Compared to those without hepatic steatosis, eight systemic inflammatory indicators were significantly correlated with hepatic steatosis. In the fully adjusted model, this correlation was slightly smaller but still significant (**Table 2**). In CRP, the risk of developing hepatic steatosis increased monotonically with increasing CRP levels, using <1.0 as a reference. In Model 1, OR= 1.00, 2.92, 5.79, 8.34, 10.65; in Model 2, OR= 1.00, 1.24, 1.43, 1.47, 1.46; in fully adjusted Model 3, OR= 1.00, 1.21, 1.34, 1.35, 1.30.

**Table 2:** Associations between systemic inflammation indicators and hepatic steatosis.

variables	Model 1	Model 2	Model 3
C-reactive protein (mg/L)			
<1.0	1.00 (ref.)	1.00 (ref.)	1.00 (ref.)
(1.0, 2.0)	2.92 (2.85~2.99)	1.24 (1.19~1.28)	1.21 (1.17~1.26)
(2.0, 3.0)	5.79 (5.64~5.95)	1.43 (1.37~1.49)	1.34 (1.29~1.40)
(3.0, 4.0)	8.34 (8.10~8.58)	1.47 (1.41~1.54)	1.35 (1.29~1.41)
>=4.0	10.65 (10.37~10.95)	1.46 (1.40~1.53)	1.30 (1.24~1.36)
Lymphocyte count (10^9cells/L)			
<1.0	0.73 (0.71~0.74)	0.91 (0.88~0.94)	0.90 (0.87~0.93)
(1.0, 2.0)	1.00 (ref.)	1.00 (ref.)	1.00 (ref.)
(2.0, 3.0)	1.32 (1.30~1.34)	1.10 (1.07~1.13)	1.07 (1.04~1.11)
(3.0, 4.0)	1.66 (1.63~1.70)	1.21 (1.16~1.25)	1.14 (1.09~1.18)
>=4.0	2.08 (2.02~2.14)	1.38 (1.31~1.45)	1.23 (1.16~1.29)
Monocyte count (10^9cells/L)			
<0.3	1.00 (ref.)	1.00 (ref.)	1.00 (ref.)
(0.3, 0.5)	1.23 (1.21~1.26)	1.08 (1.05~1.11)	1.09 (1.05~1.12)
(0.5, 0.7)	1.48 (1.45~1.51)	1.12 (1.08~1.15)	1.12 (1.08~1.15)
(0.7, 0.9)	1.71 (1.67~1.74)	1.14 (1.10~1.19)	1.13 (1.08~1.17)
>=0.9	2.12 (2.07~2.17)	1.22 (1.17~1.27)	1.18 (1.13~1.24)
Neutrophil count (10^9cells/L)			
<2.0	1.00 (ref.)	1.00 (ref.)	1.00 (ref.)
(2.0, 4.0)	1.34 (1.31~1.36)	1.15 (1.11~1.19)	1.07 (1.03~1.11)
(4.0, 6.0)	1.70 (1.67~1.74)	1.24 (1.20~1.28)	1.08 (1.04~1.12)
(6.0, 8.0)	1.97 (1.92~2.02)	1.29 (1.24~1.35)	1.06 (1.01~1.10)
>=8.0	2.11 (2.06~2.16)	1.36 (1.29~1.42)	1.04 (0.99~1.09)
Lymphocyte-to-monocyte ratio (LMR)			
<3.0	1.00 (ref.)	1.00 (ref.)	1.00 (ref.)
(3.0, 4.0)	1.05 (1.03~1.07)	1.04 (1.01~1.08)	1.05 (1.02~1.09)
(4.0, 5.0)	1.07 (1.05~1.09)	1.09 (1.05~1.12)	1.09 (1.05~1.13)
(5.0, 6.0)	1.09 (1.06~1.11)	1.10 (1.06~1.15)	1.09 (1.05~1.14)
>=6.0	1.11 (1.08~1.13)	1.13 (1.08~1.17)	1.09 (1.05~1.14)
Neutrophil-to-lymphocyte ratio (NLR)			
<2.0	1.00 (ref.)	1.00 (ref.)	1.00 (ref.)
(2.0, 3.0)	1.02 (1.00~1.04)	1.02 (0.99~1.06)	0.99 (0.96~1.03)
(3.0, 4.0)	1.00 (0.98~1.03)	1.00 (0.97~1.04)	0.95 (0.91~0.98)
(4.0, 5.0)	0.96 (0.94~0.98)	0.97 (0.94~1.01)	0.89 (0.86~0.93)
>=5.0	0.88 (0.86~0.90)	0.98 (0.95~1.02)	0.86 (0.82~0.89)
Platelet-to-lymphocyte ratio (PLR)			
<100	1.63 (1.60~1.66)	1.08 (1.04~1.12)	1.05 (1.01~1.08)
(100, 120)	1.36 (1.34~1.39)	1.07 (1.03~1.10)	1.07 (1.03~1.11)
(120, 150)	1.17 (1.15~1.19)	1.00 (0.97~1.04)	1.00 (0.97~1.04)
(150, 200)	1.00 (ref.)	1.00 (ref.)	1.00 (ref.)
>=200	0.82 (0.81~0.84)	0.96 (0.92~1.00)	0.95 (0.91~0.99)
Systemic immune-inflammation index (SII)			
<400	1.00 (ref.)	1.00 (ref.)	1.00 (ref.)
(400, 500)	1.01 (0.99~1.03)	1.02 (0.99~1.06)	0.99 (0.96~1.03)
(500, 600)	1.04 (1.02~1.06)	1.05 (1.02~1.09)	1.01 (0.97~1.04)
(600, 800)	1.05 (1.02~1.06)	1.06 (1.02~1.09)	0.98 (0.94~1.01)
>=800	0.99 (0.97~1.01)	1.07 (1.04~1.11)	0.95 (0.91~0.98)

Values were presented as odds ratios (95% confidence interval).

### 3.3 Prospective study

**Figure 2:**
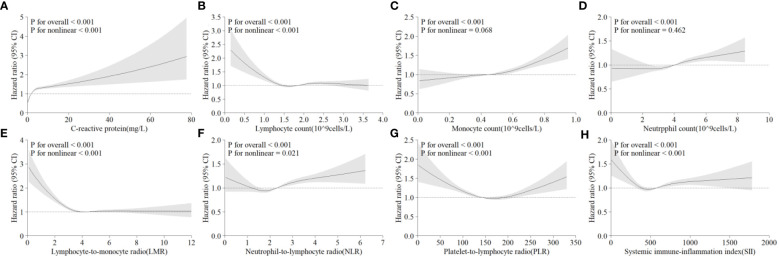
Multivariable-adjusted associations between different systematic information indicators and the risk of NAFLD according to restricted cubic spline regression. **(A)** C-reactive protein; **(B)** lymphocyte count; **(C)** monocyte count; **(D)** neutrophil count; **(E)** lymphocyte-to-monocyte ratio (lymphocytes/monocytes); **(F)** neutrophil-to-lymphocyte ratio (neutrophils/lymphocytes); **(G)** platelet-to-lymphocyte ratio (platelets/lymphocytes); **(H)** systemic immune-inflammation index (neutrophils × platelets/lymphocytes). HR, hazard ratio; CI, confidence interval.

After removing participants who had hepatic steatosis and NAFLD at baseline, a total of 4,145 of the remaining participants were diagnosed with NAFLD over a mean follow-up of 13.9 years. In Model 3, CRP levels were significantly and positively associated with the risk of developing NAFLD (**Figure 2A**). In comparison to individuals with CRP <1.0 mg/L, those with CRP >4.0 mg/L had a 2.18% greater risk of NAFLD (95% CI: 1.94–2.45) (**Table 3**). A linear positive correlation was found between monocyte count, neutrophil count, and incident NAFLD. Nevertheless, a nonsignificant linear trend was observed for lower neutrophil and monocyte counts (**Figures 2C**, **D**). L-shaped relationships were observed among lymphocyte count, the LMR, and the occurrence of NAFLD, with a decreasing trend at higher lymphocyte counts and LMR; however, these relationships were not statistically significant (**Figures 2B**, **E**). The incidence of NAFLD showed a U-shaped nonlinear correlation with the NLR, PLR, and SII (**Figures 2F**–**H**). For instance, in the PLR subgroup, the <100 group had the highest HR for the occurrence of NAFLD, and the HR (95% CI) was 1.24 (1.13~1.37) for the PLR subgroup compared with the 150–200 subgroup (**Table 3**).

**Table 3:** Associations between systemic inflammation indicators and NAFLD.

variables	Model 1	Model 2	Model 3
C-reactive protein (mg/L)			
<1.0	1.00 (ref.)	1.00 (ref.)	1.00 (ref.)
(1.0, 2.0)	1.52 (1.38~1.67)	1.32 (1.20~1.45)	1.30 (1.18~1.43)
(2.0, 3.0)	2.09 (1.88~2.32)	1.66 (1.49~1.85)	1.61 (1.44~1.79)
(3.0, 4.0)	2.47 (2.20~2.78)	1.85 (1.64~2.09)	1.77 (1.57~2.00)
>=4.0	3.17 (2.84~3.54)	2.29 (2.05~2.57)	2.18 (1.95~2.45)
Lymphocyte count (10^9cells/L)			
<1.0	1.09 (1.01~1.18)	1.10 (1.01~1.19)	1.10 (1.02~1.19)
(1.0, 2.0)	1.00 (ref.)	1.00 (ref.)	1.00 (ref.)
(2.0, 3.0)	1.10 (1.01~1.19)	1.05 (0.96~1.14)	1.03 (0.95~1.12)
(3.0, 4.0)	1.24 (1.11~1.38)	1.11 (0.99~1.26)	1.08 (0.97~1.20)
>=4.0	1.44 (1.25~1.65)	1.15 (0.99~1.32)	1.09 (0.94~1.25)
Monocyte count (10^9cells/L)			
<0.3	1.00 (ref.)	1.00 (ref.)	1.00 (ref.)
(0.3, 0.5)	1.11 (1.02~1.21)	1.05 (0.96~1.14)	1.04 (0.95~1.13)
(0.5, 0.7)	1.25 (1.14~1.36)	1.10 (1.01~1.20)	1.08 (0.99~1.18)
(0.7, 0.9)	1.41 (1.27~1.57)	1.17 (1.05~1.30)	1.13 (1.02~1.26)
>=0.9	1.96 (1.77~2.17)	1.47 (1.32~1.63)	1.40 (1.26~1.56)
Neutrophil count (10^9cells/L)			
<2.0	1.00 (ref.)	1.00 (ref.)	1.00 (ref.)
(2.0, 4.0)	1.19 (1.08~1.31)	1.12 (1.02~1.23)	1.09 (0.99~1.20)
(4.0, 6.0)	1.36 (1.24~1.50)	1.22 (1.10~1.34)	1.16 (1.06~1.28)
(6.0, 8.0)	1.58 (1.42~1.77)	1.34 (1.20~1.50)	1.26 (1.13~1.41)
>=8.0	1.86 (1.65~2.09)	1.47 (1.30~1.66)	1.35 (1.20~1.53)
Lymphocyte-to-monocyte ratio (LMR)			
<3.0	1.00 (ref.)	1.00 (ref.)	1.00 (ref.)
(3.0, 4.0)	0.75 (0.69~0.82)	0.80 (0.74~0.88)	0.81 (0.74~0.88)
(4.0, 5.0)	0.69 (0.63~0.76)	0.77 (0.70~0.84)	0.77 (0.70~0.85)
(5.0, 6.0)	0.69 (0.62~0.76)	0.77 (0.70~0.86)	0.78 (0.70~0.87)
>=6.0	0.69 (0.63~0.77)	0.77 (0.69~0.86)	0.77 (0.70~0.86)
Neutrophil-to-lymphocyte ratio (NLR)			
<2.0	1.00 (ref.)	1.00 (ref.)	1.00 (ref.)
(2.0, 3.0)	0.91 (0.82~1.01)	0.91 (0.83~1.01)	0.91 (0.8~1.00)
(3.0, 4.0)	0.95 (0.86~1.05)	0.94 (0.85~1.04)	0.93 (0.84~1.02)
(4.0, 5.0)	1.15 (1.03~1.28)	1.12 (1.01~1.25)	1.10 (0.99~1.22)
>=5.0	1.29 (1.17~1.42)	1.21 (1.10~1.34)	1.18 (1.07~1.30)
Platelet-to-lymphocyte ratio (PLR)			
<100	1.45 (1.32~1.59)	1.24 (1.13~1.36)	1.21 (1.18~1.33)
(100, 120)	1.19 (1.08~1.31)	1.10 (1.00~1.22)	1.10 (1.00~1.21)
(120, 150)	1.07 (0.98~1.17)	1.03 (0.94~1.13)	1.03 (0.94~1.12)
(150, 200)	1.00 (ref.)	1.00 (ref.)	1.00 (ref.)
>=200	1.14 (1.03~1.27)	1.16 (1.04~1.28)	1.15 (1.04~1.28)
Systemic immune-inflammation index (SII)			
<400	1.00 (ref.)	1.00 (ref.)	1.00 (ref.)
(400, 500)	0.92 (0.84~1.02)	0.93 (0.85~1.02)	0.92 (0.84~1.01)
(500, 600)	0.98 (0.89~1.08)	0.98 (0.89~1.08)	0.97 (0.88~1.07)
(600, 800)	0.97 (0.90~1.08)	0.98 (0.90~1.08)	0.96 (0.88~1.05)
>=800	1.19 (1.09~1.30)	1.16 (1.06~1.27)	1.12 (1.05~1.22)

Values were presented as hazard ratios (95% confidence interval).

### 3.4 Subgroup analysis

To assess the potential impact of baseline sex, age, and BMI on the occurrence of NAFLD, we further performed subgroup analyses by baseline sex group, age group (<60 vs. ≥60 years), and BMI group (<25 and ≥25) (Additional File 1: **Supplementary Table S1**). Monocyte counts showed significant interaction effects with sex (*P* for interaction <0.001), age (*P* for interaction <0.001) and BMI (*P* for interaction =0.005). This finding suggested that the neutrophil count is more strongly associated with NAFLD in older male individuals with a higher BMI. Additionally, we discovered that there was an interaction effect between the LMR and age (age<60: HR=0.98, 95% CI=0.97–1.00; age≥60: HR=0.95, 95% CI=0.93–0.97, *P* for interaction =0.018), implying that the LMR is more strongly associated with NAFLD in younger age groups.

### 3.5 Sensitivity analysis

After excluding patients who developed NAFLD at two and five years of follow-up, and adjusting for frequency of alcohol consumption, the results remained virtually robust (Additional File 1: **Supplementary Table S2**).

## 4 Discussion

In the present study, the levels of systemic inflammatory indicators were significantly correlated with hepatic steatosis, and there were both linear and nonlinear relationships between those indicators and NAFLD: a nonlinear positive correlation of CRP, a linear positive correlation of monocyte count and neutrophil count, a nonlinear U-shaped correlation of NLR, PLR, and SII, and a nonlinear L-shaped correlation of lymphocyte count and LMR. These correlations remained even after complete adjustment for covariates. These trends were further validated in different populations by subgroup analysis and sensitivity analysis.

Previous studies have shown that CRP is essential for the development of NAFLD (19). However, we validated the exact nonlinear correlation between CRP and NAFLD in a larger population, identifying CRP as a key predictor of NAFLD risk. Crucially, this nonlinear correlation remained robust across subgroups and sensitivity analyses, even after adjusting for all potential confounders, and was most prominent among the selected indicators of inflammation. Recent studies have shown that CRP, a traditional nonspecific acute phase protein produced by the liver that upregulates NF-kB activity and contributes to pathways that interfere with insulin signalling, is one of the inflammatory cytokines associated with NAFLD (37–39).

There is currently a dearth of research on the possible correlation between variations in leukocyte counts and the risk of NAFLD. Furthermore, a thorough understanding of how T-cell subsets cooperate and become activated in NAFLD, as opposed to NASH, to enhance hepatic inflammation is lacking. The majority of the literature that is currently available concentrates on T-cell subsets as opposed to the cytokines that T cells produce. Here, after removing outliers, we confirmed an L-shaped correlation between lymphocyte counts and the development of NAFLD (40), as well as a linear positive correlation between neutrophil counts, monocyte counts, and the occurrence of NAFLD.

In contrast to their beneficial role during infections, neutrophils typically have detrimental effects on chronic inflammatory diseases due to their production of reactive oxygen species, cytokines, proteases, and neutrophil extracellular traps (NETs) (41, 42). Several studies in humans and mice have emphasized the role of neutrophils in the development of NAFLD. Neutrophils promote the activation of c-Jun N-terminal kinase (JNK) and reduce the hepatic expression of fibroblast growth factor 21. Thus, neutrophil depletion decreases Bmal1 expression and the circadian locomotor output cycles kaput (CLOCK) and reduces overall JNK activation, decreasing hepatic steatosis (43). In mouse models, neutrophil proteases can be inhibited to decrease the recruitment and infiltration of neutrophils, halting the course of NAFLD (44). Previous research has indicated that neutrophils can control monocyte and macrophage inflammatory responses, starting from the point at which they are recruited and ending when inflammation finally returns to baseline. The recruitment of neutrophils to the site of damage during the inflammatory response might affect the inflammatory activity of recruited monocyte and macrophage populations, hence regulating the severity of the inflammatory response. Apoptotic neutrophils further control the response of macrophage and monocyte populations and promote continuous reprogramming of the immune system against inflammation, which is essential for tissue healing and inflammation reduction (45). The positive correlation trend found in this study between neutrophil count, monocyte count, and the occurrence of NAFLD confirms the findings of previous studies.

The LMR, NLR, PLR, and SII are composite inflammatory indicators derived from white blood cell counts and platelet ratios. Compared to counting only white blood cells, which are thought to more accurately represent the degree of systemic inflammation (11, 12, 26, 46), our findings confirm this theory. According to the subgroup and sensitivity analyses, the composite inflammatory indicators showed a more significant correlation with NAFLD than did simple blood cell counts. However, previous research on the relationship between the SII and NAFLD has shown some inconsistencies with our findings. Several studies from the NHANES database have presented different results. One study involving 6792 adults revealed that the SII was inversely associated with the controlled attenuation parameter (CAP), which measures the degree of hepatic steatosis, and showed no significant correlation with liver stiffness measurement (LSM) for fibrosis grading (47). Another 5.6-year follow-up study revealed a direct nonlinear association between the Log2-SII and overall mortality in NAFLD patients (48). However, in this large cohort study, we have presented compelling results and shown the precise association between these composite indicators and NAFLD. The majority of the correlation curves followed a U-shaped pattern. Additionally, the SII is a widely available, user-friendly, cost-effective, noninvasive technique with potential therapeutic advantages (12).

Infiltrated leukocytes produce inflammatory cytokines that stimulate hepatic immune cells, leading to increased hepatic inflammation and contributing to the progression from simple steatosis to non-alcoholic steatohepatitis (NASH) and eventually to fibrosis (49). Chemokine systems not only attract immune cells but also directly stimulate hepatocytes and hepatic stellate cells, enhancing their activities. Studies have shown elevated expression of chemokines and their receptors in the livers of obese patients with advanced steatosis and NASH (50). Inflammation plays a crucial role in the progression of NAFLD, cytokines and chemokines may have multiple important roles in its pathogenesis, influencing the predictive indices of NAFLD. Further research on the role of these molecules could provide valuable insights into the predictive indices of NAFLD (51).

NAFLD is a widespread chronic liver disease with a complex cause. Among these factors, inflammation is believed to be a crucial element in driving the progression from simple fatty liver to more severe forms of liver injury, including steatohepatitis, cirrhosis, and hepatocellular carcinoma. Although several inflammatory mediators have been identified in earlier research, the critical factors accelerating the development of NAFLD remain unclear and may differ among patients (52). Given the characterization of systemic inflammation indicators as continuous variables, it is challenging to pinpoint the precise percentage of the population at increased risk of NAFLD due to inflammation in the clinical setting. Our research provides reference risk values for NAFLD incidence, which can be useful indicators for the creation of NAFLD prediction models. As inflammation plays a crucial role in the development of NAFLD, anti-inflammatory drugs may improve the course of hepatic steatosis. In light of our findings, early intervention for systemic inflammation could be a viable treatment to lower the incidence of NAFLD.

## 5 Strengths and limitations

The UKB is a sizable prospective cohort including diverse inflammatory indicators for over 500,000 individuals with nearly 14 years of follow-up. This makes our study the largest analysis to date exploring the correlation between systemic inflammatory indicators and NAFLD. Notably, the study integrated a composite measure of systemic inflammatory indicators, including CRP and differential leukocyte count, and composite measures such as the LMR, NLR, PLR, and SII. In addition, the study employed a stringent exclusion criterion, setting a threshold of HSI>36, to exclude participants who may have had NAFLD at baseline.

However, this study has several limitations. First, as an observational study, a causal relationship between various systemic inflammatory indicators and NAFLD could not be established. Second, the evaluation of confounding factors and inflammatory indicators was limited to the use of the baseline data, leaving out pertinent data from the follow-up that may have evolved but was not recorded or examined. Furthermore, despite adjusting for key confounders, bias due to unknown and unmeasured confounders may persist. Third, the determination of NAFLD risk relies on admission and death records, potentially leading to an underestimation of the true incidence. Fourth, the study population primarily consisted of white British individuals, with a smaller representation from nonwhite ethnic groups, thereby limiting the generalizability of the findings to other ethnic populations. Fifth, this aspect of durg use was not considered in our study due to the limited information on substance use in the UK Biobank. Finally, considering the insulting nature of the terms ‘nonalcoholic’ and ‘fatty’ in NAFLD, the latest Delphi consensus statement replaces NAFLD with metabolic dysfunction associated steatotic liver disease (MASLD), defined as the presence of hepatic steatosis in conjunction with one cardiometabolic risk factor (CMRF) (53). However, NAFLD was used in this study, and not all cardiometabolic risk factor data were included; only hypertension, diabetes, high triglycerides, and low HDL were included as covariates.

## 6 Conclusion

In conclusion, systemic inflammation levels are significantly associated with hepatic steatosis and NAFLD risk, with CRP having the strongest correlation and composite systemic inflammatory indicators being more strongly correlated than any individual blood cell count variable. Given the high prevalence of NAFLD and the resulting global health burden, early prevention of inflammation is essential. This work contributes to the creation of a reference index for future NAFLD occurrence prediction models. In addition, future studies need to demonstrate causality and investigate other interventions that may be effective in reducing the burden of NAFLD.

## Data availability statement

Publicly available datasets were analyzed in this study. This data can be found here: https://biobank.ndph.ox.ac.uk/showcase/browse.cgi?id=-2&cd=download.

## Author contributions

HG: Conceptualization, Data curation, Formal analysis, Funding acquisition, Investigation, Methodology, Project administration, Resources, Software, Supervision, Validation, Visualization, Writing – original draft, Writing – review & editing. QH: Data curation, Methodology, Software, Supervision, Validation, Writing – review & editing. LZ: Project administration, Supervision, Writing – review & editing. ZF: Investigation, Methodology, Software, Supervision, Validation, Writing – review & editing. MS: Investigation, Methodology, Software, Validation, Writing – review & editing. JJ: Conceptualization, Data curation, Formal analysis, Writing – original draft. XY: Data curation, Funding acquisition, Project administration, Writing – original draft. YS: Conceptualization, Data curation, Formal analysis, Methodology, Project administration, Resources, Software, Supervision, Writing – original draft, Writing – review & editing. JD: Conceptualization, Data curation, Project administration, Writing – original draft, Writing – review & editing.

## References

[1] Younossi ZM Golabi P Paik JM Henry A Van Dongen C Henry L. The global epidemiology of nonalcoholic fatty liver disease (NAFLD) and nonalcoholic steatohepatitis (NASH): a systematic review. Hepatol (Baltimore Md). (2023) 77:1335–47. doi: 10.1097/HEP.0000000000000004 PMC10026948 36626630

[2] Li W Alazawi W. Non-alcoholic fatty liver disease. Clin Med (London England). (2020) 20:509–12. doi: 10.7861/clinmed.2020-0696 PMC7539730 32934047

[3] Ekstedt M Hagström H Nasr P Fredrikson M Stål P Kechagias S. Fibrosis stage is the strongest predictor for disease-specific mortality in NAFLD after up to 33 years of follow-up. Hepatol (Baltimore Md). (2015) 61:1547–54. doi: 10.1002/hep.27368 25125077

[4] Taylor RS Taylor RJ Bayliss S Hagström H Nasr P Schattenberg JM. Association between fibrosis stage and outcomes of patients with nonalcoholic fatty liver disease: A systematic review and meta-analysis. Gastroenterology. (2020) 158:1611–25.e12. doi: 10.1053/j.gastro.2020.01.043 32027911

[5] Younossi ZM Stepanova M Younossi Y Golabi P Mishra A Rafiq N. Epidemiology of chronic liver diseases in the USA in the past three decades. Gut. (2020) 69:564–8. doi: 10.1136/gutjnl-2019-318813 31366455

[6] Riazi K Azhari H Charette JH Underwood FE King JA Afshar EE. The prevalence and incidence of NAFLD worldwide: a systematic review and meta-analysis. Lancet Gastroenterol Hepatol. (2022) 7:851–61. doi: 10.1016/S2468-1253(22)00165-0 35798021

[7] Younossi ZM Blissett D Blissett R Henry L Stepanova M Younossi Y. The economic and clinical burden of nonalcoholic fatty liver disease in the United States and Europe. Hepatol (Baltimore Md). (2016) 64:1577–86. doi: 10.1002/hep.28785 27543837

[8] Iyengar NM Gucalp A Dannenberg AJ Hudis CA. Obesity and cancer mechanisms: tumor microenvironment and inflammation. J Clin Oncol. (2016) 34:4270–6. doi: 10.1200/JCO.2016.67.4283 PMC5562428 27903155

[9] Kronsten VT Tranah TH Pariante C Shawcross DL. Gut-derived systemic inflammation as a driver of depression in chronic liver disease. J hepatol. (2022) 76:665–80. doi: 10.1016/j.jhep.2021.11.008 34800610

[10] Cao W Cao Z Tian Y Zhang L Wang W Tang L. Neutrophils are associated with higher risk of incident amyotrophic lateral sclerosis in a BMI- and age-dependent manner. Ann neurol. (2023) 94:942–54. doi: 10.1002/ana.26760 37554051

[11] Nøst TH Alcala K Urbarova I Byrne KS Guida F Sandanger TM. Systemic inflammation markers and cancer incidence in the UK Biobank. Eur J Epidemiol. (2021) 36:841–8. doi: 10.1007/s10654-021-00752-6 PMC8416852 34036468

[12] Yang X Zhao S Wang S Cao X Xu Y Yan M. Systemic inflammation indicators and risk of incident arrhythmias in 478,524 individuals: evidence from the UK Biobank cohort. BMC Med. (2023) 21:76. doi: 10.1186/s12916-023-02770-5 36855116 PMC9976398

[13] Haukeland JW Damås JK Konopski Z Løberg EM Haaland T Goverud I. Systemic inflammation in nonalcoholic fatty liver disease is characterized by elevated levels of CCL2. J Hepatol. (2006) 44(6):1167–74. doi: 10.1016/j.jhep.2006.02.011 16618517

[14] Fontes-Cal TCM Mattos RT Medeiros NI Pinto BF Belchior-Bezerra M Roque-Souza B. Crosstalk between plasma cytokines, inflammation, and liver damage as a new strategy to monitoring NAFLD progression. Front Immunol. (2021) 12:708959. doi: 10.3389/fimmu.2021.708959 34447378 PMC8383065

[15] Hu B Yang XR Xu Y Sun YF Sun C Guo W. Systemic immune-inflammation index predicts prognosis of patients after curative resection for hepatocellular carcinoma. Clin Cancer Res. (2014) 20:6212–22. doi: 10.1158/1078-0432.CCR-14-0442 25271081

[16] Mahemuti N Jing X Zhang N Liu C Li C Cui Z. Association between systemic immunity-inflammation index and hyperlipidemia: A population-based study from the NHANES (2015–2020). Nutrients. (2023) 15:1177. doi: 10.3390/nu15051177 36904176 PMC10004774

[17] Liu B Wang J Li YY Li KP Zhang Q. The association between systemic immune-inflammation index and rheumatoid arthritis: evidence from NHANES 1999–2018. Arthritis Res Ther. (2023) 25:34. doi: 10.1186/s13075-023-03018-6 36871051 PMC9985219

[18] Song Y Guo W Li Z Guo D Li Z Li Y. Systemic immune-inflammation index is associated with hepatic steatosis: Evidence from NHANES 2015–2018. Front Immunol. (2022) 13:1058779. doi: 10.3389/fimmu.2022.1058779 36466832 PMC9718528

[19] Duan Y Pan X Luo J Xiao X Li J Bestman PL. Association of inflammatory cytokines with non-alcoholic fatty liver disease. Front Immunol. (2022) 13:880298. doi: 10.3389/fimmu.2022.880298 35603224 PMC9122097

[20] Van Herck MA Weyler J Kwanten WJ Dirinck EL De Winter BY Francque SM. The differential roles of T cells in non-alcoholic fatty liver disease and obesity. Front Immunol. (2019) 10:82. doi: 10.3389/fimmu.2019.00082 30787925 PMC6372559

[21] Barrow F Revelo XS. The B side of B cells in NAFLD. Hepatol (Baltimore Md). (2022) 76:914–6. doi: 10.1002/hep.32481 PMC9489625 35340064

[22] Lee YJ Lee HR Shim JY Moon BS Lee JH Kim JK. Relationship between white blood cell count and nonalcoholic fatty liver disease. Digest liver Dis. (2010) 42:888–94. doi: 10.1016/j.dld.2010.04.005 20472517

[23] Collins R. What makes UK Biobank special? Lancet (London England). (2012) 379:1173–4. doi: 10.1016/S0140-6736(12)60404-8 22463865

[24] Gong P Liu Y Gong Y Chen G Zhang X Wang S. The association of neutrophil to lymphocyte ratio, platelet to lymphocyte ratio, and lymphocyte to monocyte ratio with post-thrombolysis early neurological outcomes in patients with acute ischemic stroke. J neuroinflamm. (2021) 18:51. doi: 10.1186/s12974-021-02090-6 PMC7896410 33610168

[25] Kriplani A Pandit S Chawla A de la Rosette J Laguna P Jayadeva Reddy S. Neutrophil-lymphocyte ratio (NLR), platelet-lymphocyte ratio (PLR) and lymphocyte-monocyte ratio (LMR) in predicting systemic inflammatory response syndrome (SIRS) and sepsis after percutaneous nephrolithotomy (PNL). Urolithiasis. (2022) 50:341–8. doi: 10.1007/s00240-022-01319-0 PMC9110452 35246692

[26] Zhang YR Wang JJ Chen SF Wang HF Li YZ Ou YN. Peripheral immunity is associated with the risk of incident dementia. Mol Psychiatry. (2022) 27:1956–62. doi: 10.1038/s41380-022-01446-5 35079124

[27] Lee JH Kim D Kim HJ Lee CH Yang JI Kim W. Hepatic steatosis index: a simple screening tool reflecting nonalcoholic fatty liver disease. Digest liver Dis. (2010) 42:503–8. doi: 10.1016/j.dld.2009.08.002 19766548

[28] Li W Xiao H Wu H Pan C Deng K Xu X. Analysis of environmental chemical mixtures and nonalcoholic fatty liver disease: NHANES 1999–2014. Environ pollut (Barking Essex: 1987). (2022) 311:119915. doi: 10.1016/j.envpol.2022.119915 35970346

[29] Castera L Friedrich-Rust M Loomba R. Noninvasive assessment of liver disease in patients with nonalcoholic fatty liver disease. Gastroenterology. (2019) 156:1264–81.e4. doi: 10.1053/j.gastro.2018.12.036 30660725 PMC7505052

[30] Petermann-Rocha F Wirth MD Boonpor J Parra-Soto S Zhou Z Mathers JC. Associations between an inflammatory diet index and severe non-alcoholic fatty liver disease: a prospective study of 171,544 UK Biobank participants. BMC Med. (2023) 21:123. doi: 10.1186/s12916-023-02793-y 37013578 PMC10071692

[31] Chen L Fan Z Lv G. Associations of muscle mass and grip strength with severe NAFLD: A prospective study of 333,295 UK Biobank participants. J hepatol. (2022) 77:1453–4. doi: 10.1016/j.jhep.2022.05.005 35605742

[32] Huang H Liu Z Xie J Xu C. Association between night shift work and NAFLD: a prospective analysis of 281,280 UK Biobank participants. BMC Public Health. (2023) 23:1282. doi: 10.1186/s12889-023-16204-7 37400787 PMC10318710

[33] Hagström H Adams LA Allen AM Byrne CD Chang Y Grønbaek H. Administrative coding in electronic health care record-based research of NAFLD: an expert panel consensus statement. Hepatol (Baltimore Md). (2021) 74:474–82. doi: 10.1002/hep.31726 PMC8515502 33486773

[34] Harrison S Davies AR Dickson M Tyrrell J Green MJ Katikireddi SV. The causal effects of health conditions and risk factors on social and socioeconomic outcomes: Mendelian randomization in UK Biobank. Int J Epidemiol. (2020) 49:1661–81. doi: 10.1093/ije/dyaa114 PMC7746412 32808034

[35] Kassi E Pervanidou P Kaltsas G Chrousos G. Metabolic syndrome: definitions and controversies. BMC Med. (2011) 9:48. doi: 10.1186/1741-7015-9-48 21542944 PMC3115896

[36] Liu X Zhang D Liu Y Sun X Han C Wang B. Dose-response association between physical activity and incident hypertension: A systematic review and meta-analysis of cohort studies. Hypertens (Dallas Tex: 1979). (2017) 69:813–20. doi: 10.1161/HYPERTENSIONAHA.116.08994 28348016

[37] Barretto JR Boa-Sorte N Vinhaes CL Malta-Santos H Rebouças-Silva J Ramos CF. Heightened plasma levels of transforming growth factor beta (TGF-β) and increased degree of systemic biochemical perturbation characterizes hepatic steatosis in overweight pediatric patients: A cross-sectional study. Nutrients. (2020) 12:1650. doi: 10.3390/nu12061650 32498337 PMC7352859

[38] Bian F Yang XY Xu G Zheng T Jin S. CRP-induced NLRP3 inflammasome activation increases LDL transcytosis across endothelial cells. Front Pharmacol. (2019) 10:40. doi: 10.3389/fphar.2019.00040 30761006 PMC6363700

[39] Khan RS Bril F Cusi K Newsome PN. Modulation of insulin resistance in nonalcoholic fatty liver disease. Hepatol (Baltimore Md). (2019) 70:711–24. doi: 10.1002/hep.30429 30556145

[40] Huby T Gautier EL. Immune cell-mediated features of non-alcoholic steatohepatitis. Nat Rev Immunol. (2022) 22:429–43. doi: 10.1038/s41577-021-00639-3 PMC8570243 34741169

[41] Jorch SK Kubes P. An emerging role for neutrophil extracellular traps in noninfectious disease. Nat Med. (2017) 23:279–87. doi: 10.1038/nm.4294 28267716

[42] Soehnlein O Steffens S Hidalgo A Weber C. Neutrophils as protagonists and targets in chronic inflammation. Nat Rev Immunol. (2017) 17:248–61. doi: 10.1038/nri.2017.10 28287106

[43] Crespo M Gonzalez-Teran B Nikolic I Mora A Folgueira C Rodríguez E. Neutrophil infiltration regulates clock-gene expression to organize daily hepatic metabolism. eLife. (2020) 9:59258. doi: 10.7554/eLife.59258 PMC7723411 33287957

[44] Herrero-Cervera A Soehnlein O Kenne E. Neutrophils in chronic inflammatory diseases. Cell Mol Immunol. (2022) 19:177–91. doi: 10.1038/s41423-021-00832-3 PMC8803838 35039631

[45] Marwick JA Mills R Kay O Michail K Stephen J Rossi AG. Neutrophils induce macrophage anti-inflammatory reprogramming by suppressing NF-κB activation. Cell Death dis. (2018) 9:665. doi: 10.1038/s41419-018-0710-y 29867198 PMC5986789

[46] Valero C Lee M Hoen D Weiss K Kelly DW Adusumilli PS. Pretreatment neutrophil-to-lymphocyte ratio and mutational burden as biomarkers of tumor response to immune checkpoint inhibitors. Nat Commun. (2021) 12:729. doi: 10.1038/s41467-021-20935-9 33526794 PMC7851155

[47] Xie R Xiao M Li L Ma N Liu M Huang X. Association between SII and hepatic steatosis and liver fibrosis: A population-based study. Front Immunol. (2022) 13:925690. doi: 10.3389/fimmu.2022.925690 36189280 PMC9520084

[48] Zhao E Cheng Y Yu C Li H Fan X. The systemic immune-inflammation index was non-linear associated with all-cause mortality in individuals with nonalcoholic fatty liver disease. Ann Med. (2023) 55:2197652. doi: 10.1080/07853890.2023.2197652 37052341 PMC10115001

[49] Roh YS Seki E. Chemokines and chemokine receptors in the development of NAFLD. Adv Exp Med Biol. (2018) 1061:45–53. doi: 10.1007/978-981-10-8684-7_4 29956205

[50] Bertola A Bonnafous S Anty R Patouraux S Saint-Paul MC Iannelli A. Hepatic expression patterns of inflammatory and immune response genes associated with obesity and NASH in morbidly obese patients. PloS One. (2010) 5:e13577. doi: 10.1371/journal.pone.0013577 21042596 PMC2962651

[51] Lalor PF Faint J Aarbodem Y Hubscher SG Adams DH. The role of cytokines and chemokines in the development of steatohepatitis. Semin liver dis. (2007) 27:173–93. doi: 10.1055/s-2007-979470 17520517

[52] Wang H Mehal W Nagy LE Rotman Y. Immunological mechanisms and therapeutic targets of fatty liver diseases. Cell Mol Immunol. (2021) 18:73–91. doi: 10.1038/s41423-020-00579-3 33268887 PMC7852578

[53] Rinella ME Lazarus JV Ratziu V Francque SM Sanyal AJ Kanwal F. A multisociety Delphi consensus statement on new fatty liver disease nomenclature. J Hepatol. (2023) 79:1542–56. doi: 10.1016/j.aohep.2023.101133 37364790

